# A Case of Meningo-Cerebritis

**Published:** 1849-03

**Authors:** Thomas C. Osborne

**Affiliations:** Erie, Alabama


ART. IV.—*A Case of Meningo-Cerebritis.* By DR. THOMAS C. OSBORNE, of Erie, Alabama.

I was called, on the 30th of November, 1847, to prescribe for Esther, a black woman, 35 years of age, on account of what was thought to be a troublesome headache, of which she had been complaining for more than a month, but which, until three or four days previously had not compelled her to take her bed. It had been noticed that there was a gradually increasing tendency to moroseness and mental imbecility, but as she was lately purchased much of her complainings were attributed to a stubborn disposition to resist authority.

I found her in the following condition: Patient shudders if exposed to the air; stupid, but when roused answers intelligibly in the recumbent, but confusedly in the sitting, posture; surface pleasantly warm and moist; pulse 70, soft, and of ordinary volume; respiration natural; tongue clean; eyes heavy, and pupils dilated and immovable; hearing good; complains of violent pain in the head, and in the lower part of the abdomen, without soreness; urinary and intestinal functions healthy.

Prescription. V.S., and cups to the temples largely; blisters to nape and ancles, and free purgation with calomel.

Dec. 3d. Have visited patient daily, and although prompt and energetic treatment has been kept up, no change has occurred until this morning. Pulse now frequent, small, and quick; subsultus tendinum; respiration noisy; eyes lustreless; coma; involuntary dejections; perspiration profuse; very slight emaciation. She died on the 5th without convulsions.

*Sectio-cadaveris, eighteen hours after death.* Dura mater of a dull leaden color; tough adhesion between it and subjacent tunic, about two inches in length, at the summit edge of the anterior lobes of each hemisphere. Sinus loaded with black fluid blood. Arterial and venous distension extensive in the arachnoid and pia mater. Spots of various sizes of diffuse redness were scattered freely on the arachnoid at the anterior surface of the brain. Cerebrum and cerebellum quite firm; fibrous and granular texture of both densely speckled with red indelible punctuations; capillary engorgement was also every where present. The ventricles presented the same network of vessels, and redness of the meninges generally. The medulla oblongata, pons varolii, corpus callosum, and optic couches were free from discoloration, but so soft as to pit if lightly touched with the finger.

*Remarks.* By collating the previous history and post-mortem appearances, we are enabled to pronounce this a case of acute, supervening upon chronic, inflammation of the brain and its membranes. Thus, the constant headache, increasing disturbance of the intellectual functions, and mental apathy, together with the softness of certain parts of the brain, indicate chronic action, while intensely acute disease is manifested in the punctuated vascular and turgid condition of the brain and its envelops.

December 30th, 1848.

